# Clinical efficacy of sacubitril-valsartan combined with acute ST-segment elevation myocardial infarction after reperfusion: A systematic review and meta-analysis

**DOI:** 10.3389/fcvm.2022.1036151

**Published:** 2022-12-01

**Authors:** Dong Zhang, Hui Wu, Di Liu, Yunzhao Li, Gang Zhou, QingZhuo Yang, YanFang Liu

**Affiliations:** ^1^Department of Cardiology, The First College of Clinical Medical Science, China Three Gorges University, Yichang, China; ^2^Institute of Cardiovascular Diseases, China Three Gorges University, Yichang, China

**Keywords:** myocardial infarction, myocardial ischemia-reperfusion injury (MIRI), acute ST-segment elevation myocardial infarction (STEMI), sacubitril-valsartan, acute myocardial infarction

## Abstract

**Background:**

Several studies have investigated the combined use of sacubitril- valsartan after reperfusion in acute ST-segment elevation myocardial infarction (STEMI). However, the sample sizes of these studies were small and their results were somewhat heterogeneous. To determine the effect of sacubitril-valsartan on myocardial ischemia-reperfusion.

**Methods:**

Search PubMed, EMbase, Web of Science and The Cochrane Library, CNKI database, VIP database and Wanfang digital journal full-text database for eligible articles from their date of inception up to April, 2022. All data were meta-analyzed using Review Manager 5.3 and STATA 16.0 software.

**Results:**

A total of 23 studies including 2,326 patients with acute STEMI were included. These results of this meta-analysis indicated that left ventricular ejection fractions (LVEF) value within 6 months after surgery (OR, 4.29; 95% confidence interval, 3.78–4.80; *P* < 0.00001), left ventricular end-diastolic diameter (LVEDD) value within 6 months after surgery (OR, −3.11; 95% CI, −3.87 to −2.35; *P* < 0.00001) and left ventricular end-diastolic volume (LVEDV) value 6 months after operation (OR, −6.22; 95% CI, −7.10 to −5.35; *P* < 0.00001) are better than without sacubitril and valsartan.

**Conclusion:**

To sum up the above, the results of this study suggest that sacubitril- valsartan can reduce the reperfusion injury of ischemic myocardium by improving cardiac function within a follow-up period of 6 months.

## Introduction

Acute myocardial infarction (AMI) has become the leading cause of death in patients with cardiovascular disease (1). ST-segment elevation myocardial infarction (STEMI) is the most important and common disease type of AMI (2). The crucial to the treatment of STEMI patients is to quickly open the infarct-related coronary arteries, restore the blood supply to the ischemic area, save the ischemic myocardium, reduce the size of myocardial infarction, and ultimately reduce the mortality and disability rate (3). Some studies have pointed out that the blood flow recovery of ischemic myocardium will cause re-injury to surviving myocardial cells, induce malignant arrhythmia, cardiac dysfunction, myocardial remodeling and other phenomena, which is called myocardial ischemia-reperfusion injury (MIRI) (4). Therefore, timely and effective MIRI mitigation measures have attracted more and more attention of clinicians.

Sacubitril-valsartan is the world’s first dual inhibitor of enkephalinase and angiotensin receptors, and is a new single co-crystal composed of sacubitril and valsartan in equal proportions (5). In fact, sacubitril, which is a prodrug, has no biological activity, but can be metabolized into the neprilysin (NEPI) inhibitor LBQ657, this substance can effectively hinder the metabolic decomposition of endogenous natriuretic peptides by NEPI. In addition, valsartan can target the receptors of angiotensin II, reduce the secretion of angiotensin and aldosterone, and slow down the heart damage caused by the renin-angiotensin-aldosterone system (RAAS) (6). In the previous research team, in a multi-center, double-blind randomized trial, it was found that sacubitril and valsartan can significantly reduce the expression level of B-type natriuretic peptide (BNP) in serum of patients with heart failure, this result shows that sacubitril-valsartan can improves heart function (7). In Xiong’s (8) review, it was mentioned that saxablatril and valsartan can effectively increase left ventricular ejection fractions (LVEF), slow cardiac remodelingand improve cardiac function in patients with AMI. Furthermore, in another clinical study, the application effect of sacubitril and valsartan sodium tablets in cardiac rehabilitation after percutaneous coronary intervention for AMI was analyzed and compared. Significant improvement in Left ventricular end-diastolic diameter (LVEDD) levels (9). Simultaneously, another team reviewed the remodeling parameters after coronary heart disease treatment through sacubitril-valsartan, and explained that it can significantly improve the left ventricular end-diastolic volume (LVEDV) (10). Through the above studies, it is not difficult to find that sacubitril-valsartan has a significant effect on the recovery of cardiac function. Sacubitril-valsartan can simultaneously regulate RAAS and natriuretic peptide systems, dilate blood vessels, and promote natriuresis and urination, reduce sympathetic tension and other effects, and finally achieve double cardiovascular protection.

Although relevant clinical trials have explored the role of sacubitril and valsartan in helping heart function, the guideline recommendation is low due to the small sample size of the study (11). Therefore, this Meta-analysis explored the pros and cons of using sacubitril-valsartan in combination with reperfusion therapy for STEMI patients.

## Materials and methods

### Search strategy

Search PubMed, EMbase, Web of Science and The Cochrane Library, CNKI database, VIP database and Wanfang digital journal full-text database for eligible articles from their date of inception up to April, 2022. The following keywords were used: “STEMI” AND “reperfusion” AND “sacubitril-valsartan.” At the same time, combine the respective free words to make the retrieval more accurate. In addition, to ensure the credibility of the results, we excluded reviews, animal experiments, case reports, and conference reports.

### Inclusion and exclusion criteria

Eligible studies were included in this meta-analysis if (1) all patients in the studies met the diagnosis of acute STEMI [2017 ESC Guidelines for the management of AMI in patients presenting with ST-segment elevation (12)]; (2) concomitant use of sacubitril-valsartan in eligible patients who received reperfusion therapy; (3) all ages; and (4) all studies were randomized controlled trials; The following studies were excluded if (1) Animal experiments; (2) study grouped more than two groups; (3) reviews, case reports, and conference reports; and (4) data records are not detailed.

### Outcome measures

The primary endpoint was LVEF value within 6 months after surgery. The secondary endpoints were LVEF value within 6 months after surgery and LVEDD value within 6 months after surgery. The basic characteristics of all studies are shown in Table 1.

**TABLE 1:** Characteristics of the included trials.

Study	Test/Control, n	Male/Female, n	Mean age, years	Hypertension	Diabetes	Hyperlipidemia	BMI	Treatment measures
Rezq et al. (14)	100/100	T:86/14 C:88/12	T:52 ± 9.2 C:57 ± 11.6	T:34 C:38	T:40 C:34	T:86 C:94	T:28.7 ± 4.3 C:29.2 ± 3.5	T:Sacubitril/valsartan was 50 mg twice daily orally, 2 weeks C:Ramipril was started as 5 mg once daily orally
Li and Xiong (15)	50/48	T:27/23 C:26/22	T:62.4 ± 9.8 C:62.4 ± 10.1	–	–	–	–	T:Sacubitril/valsartan was 50 mg twice daily orally, 2 weeks. C:No sacubitril/valsartan treat.
Chen et al. (9)	42/39	T:27/15 C:24/15	T:51.28 ± 6.27 C:51.30 ± 6.21	T:13 C:14	T:10 C:8	T:18 C:18	–	T: Sacubitril/valsartan 50 mg/time, 2 times/day. C:Bisoprolol 5 mg, Once daily
Gu (16)	40/40	T:22/18 C:24/16	T:62.24 ± 4.64 C:62.18 ± 5.36	T:17 C:16	T:20 C:18	T:8 C:8	–	T:Sacubitril/valsartan was 25 mg twice daily orally, 2 weeks. C:No sacubitril/valsartan treat.
Zhou et al. (17)	40/40	T:30/10 C:28/12	T:68.74 ± 7.14 C:68.89 ± 7.28	–	–	–	–	T:Sacubitril/valsartan was 50 mg twice daily orally, 2 weeks. C:No sacubitril/valsartan treat.
Wang et al. (18)	80/80	T:69/11 C:67/13	T:59.0 ± 10.3 C:58.0 ± 10.4	T:32 C:37	T:19 C:15	T:37 C:46	T:28.6 ± 6.20 C:27.80 ± 5.60	T:Sacubitril/valsartan was 25 mg twice daily orally, 2 weeks. C:No sacubitril/valsartan treat.
He et al. (19)	25/25	–	–	–	–	–	–	T:Sacubitril/valsartan treat C:No sacubitril/valsartan treat.
Cai (20)	38/38	T:23/15 C:25/13	T:67.60 ± 11.513 C:68.35 ± 9.979	T:32 C:28	T:29 C:22	T:19 C:22	–	T:Sacubitril/valsartan was 50 mg twice daily orally, 2 weeks. C:No sacubitril/valsartan treat.
Yan and Hu (21)	102/98	T:67/35 C:38/30	T:45.94 ± 13.41 C:56.45 ± 13.29	–	–	–	22.78 ± 0.85	T:Sacubitril/valsartan was 50 mg twice daily orally, 2 weeks. C:No sacubitril/valsartan treat.
Li et al. (22)	50/50	T:28/22 C:27/23	T:54.44 ± 5.98 C:54.87 ± 6.12	–	–	–	–	T:Sacubitril/valsartan was 200 mg thrice daily orally, 2 weeks. C:No sacubitril/valsartan treat.
Jian et al. (23)	40/40	T:25/15 C:22/18	T:54.95 ± 7.70 C:54.13 ± 7.98	–	–	–	–	T:Sacubitril/valsartan 24/26, 49/51, and 97/103 mg twice daily C:Valsartan 40, 80, and 160 mg twice daily
Yang et al. (24)	38/38	T:35/3 C:31/7	T:60 ± 13 C:55 ± 12	T:18 C:19	T:5 C:9	T:10 C:16	T:24.1 ± 2.3 C:24.3 ± 2.6	T:Sacubitril/valsartan was 100 mg twice daily orally, 2 weeks. C:No sacubitril/valsartan treat.
Wang and Lai (25)	30/30	T:17/13 C:19/11	T:55.67 ± 4.02 C:55.31 ± 4.14	–	–	–	–	T:Sacubitril/valsartan was 50 mg twice daily orally, 2 weeks. C:No sacubitril/valsartan treat.
Salimujiang (26)	40/40	T:29/11 C:27/13	T:63.8 ± 8.4 C:65.3 ± 8.9	T:19 C:13	T:26 C:23	T:33 C:36	–	T:Sacubitril/valsartan was 50 mg twice daily orally, 2 weeks. C:No sacubitril/valsartan treat.
Zhou et al. (27)	55/55	T:34/21 C:30/25	T:61.6 ± 9.75 C:62.9 ± 11.28	T:20 C:22	T:9 C:11	T:13 C:10	–	T:Sacubitril/valsartan was 25 mg twice daily orally, 2 weeks. C:No sacubitril/valsartan treat.
Yuan et al. (28)	42/42	T:23/19 C:22/20	T:60.37 ± 4.46 C:61.31 ± 4.14	T:21 C:25	T:12 C:14	T:24 C:21	–	T:Sacubitril/valsartan was 50 mg twice daily orally, 2 weeks. C:No sacubitril/valsartan treat.
Yan (29)	36/44	T:27/9 C:36/8	T:60.19 ± 10.08 C:57.75 ± 12.76	T:17 C:20	T:11 C:18	/	T:23.53 ± 2.82 C:25.11 ± 2.76	T:Sacubitril/valsartan was 25 mg twice daily orally, 2 weeks. C:No sacubitril/valsartan treat.
Zhao et al. (30)	47/47	T:20/27 C:26/21	T:57.03 ± 9.48 C:56.42 ± 9.27	–	–	–	–	T:Sacubitril/valsartan was 25 mg twice daily orally, 2 weeks. C:No sacubitril/valsartan treat.
Zhao (31)	62/61	T:39/23 C:40/21	T:68.21 ± 2.35 C:68.36 ± 2.29	–	–	–	–	T:Sacubitril/valsartan was 20–40 mg twice daily orally, 2 weeks. C:No sacubitril/valsartan treat.
Guo (32)	50/50	T:37/13 C:34/16	T:46.3 ± 24.2 C:48.7 ± 26.1	–	–	–	–	T:Sacubitril/valsartan was 50 mg twice daily orally, 2 weeks. C:No sacubitril/valsartan treat.
Dong et al. (33)	65/66	T:51/14 C:53/13	T:60.2 ± 9.8 C:60.4 ± 10.0	T:29 C:33	T:15 C:18	–	T:23.9 ± 3.1 C: 24.3 ± 2.5	T:Sacubitril/valsartan treat C:No sacubitril/valsartan treat.
Zhao et al. (34)	45/45	T:25/20 C:27/18	T:62.8 ± 3.9 C:62.7 ± 6.1	–	–	–	–	T:Sacubitril/valsartan treat C:No sacubitril/valsartan treat.
Ma (35)	30/30	T:18/12 C:16/14	T:62.4 ± 5.6 C:63.3 ± 6.1	–	–	–	–	T:Sacubitril/valsartan was 50 mg twice daily orally, 2 weeks. C:No sacubitril/valsartan treat.

–, No report; T, test group; C, control group.

### Data extraction and quality assessment

**FIGURE 1:**
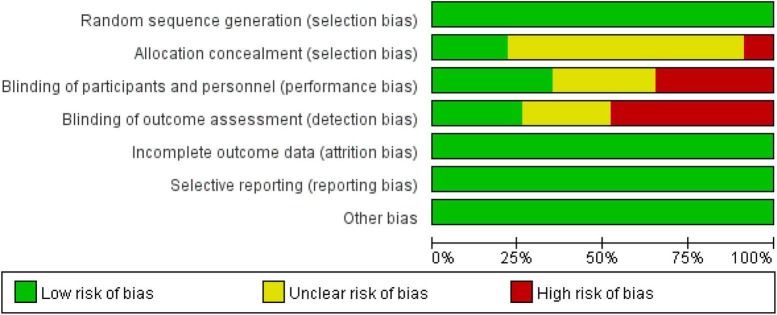
Risk of bias graph.

**FIGURE 2:**
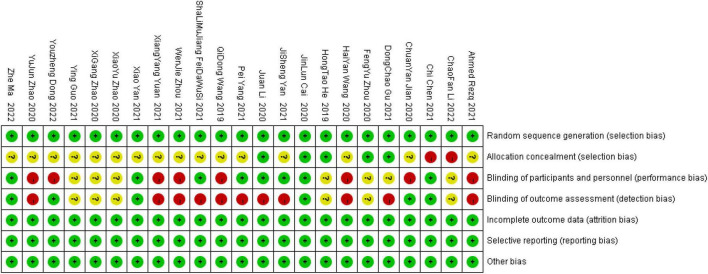
Risk of bias summary.

Two researchers independently searched the literature and extracted relevant data. If there was any disagreement during the period, they were resolved through joint discussion, and a third researcher could assist in resolving it if necessary. The methodological quality of the included studies was assessed using the “Risk of bias assessment” tool recommended in the Cochrane Handbook for the quality of the included studies in terms of random allocation method, allocation concealment, blinding, completeness of outcome data, and bias in selective reporting of findings (13). For each study, the above six items were rated as “yes” (low level of bias), “no” (highly biased), or “unclear” (lack of relevant information or uncertain bias). The bias of 23 documents is shown in Figures 1, 2.

### Statistical analysis

Review Manager 5.3 and STATA 16.0 software provided by Cochrane Network were used. For binary variables, odds ratio (OR)was used as the efficacy analysis statistic, and for numerical variables, the weighted mean difference (WMD) was used as the efficacy analysis statistic, and each effect size was expressed as a 95% confidence interval (95% CI). The chi-square test was used to analyze the heterogeneity among the studies. When there was a high degree of statistical heterogeneity among studies (*P* < 0.1, *I*^2^ > 50%), a random-effects model was used for analysis, otherwise, a fixed-effects model was used. The SMD is calculated as the difference between the estimated means of the two groups divided by the standard deviation of the mean, and is used for data with different measures or units of measurement for the outcome variable, thereby eliminating the effect of dimension. Publication bias was assessed qualitatively by funnel plots and quantitatively by Egger’s linear regression method. A two-sided *P* < 0.05 was considered statistically significant.

## Results

### Characteristics of the included studies

**FIGURE 3:**
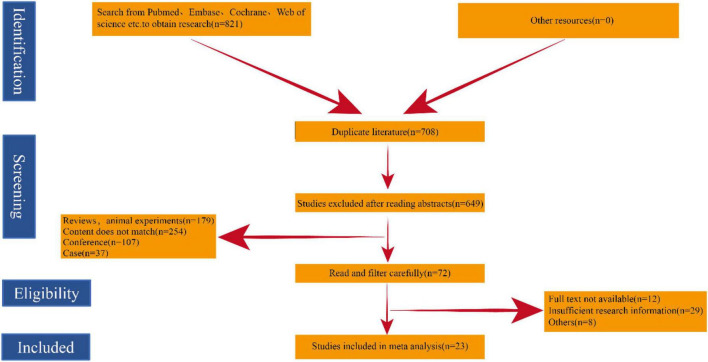
Flow diagram of study selection.

Through our search, we initially screened a total of 821 potential studies. After excluding duplicates, a total of 708 papers were checked. After careful review, 23 studies met the inclusion criteria and were included in our meta-analysis. The overall search strategy flow chart is shown in Figure 3.

As shown in Table 1, a total of 23 studies (9, 14–35) involving 2,326 patients were included in the meta-analysis. In the 23 studies included, it was mentioned that the baselines of the experimental group and the control group were similar and comparable. All studies refer to random words. All studies included in the meta-analysis were randomized controlled trials. Table 1 summarizes the main characteristics of each study.

### Major clinical outcomes

**FIGURE 4:**
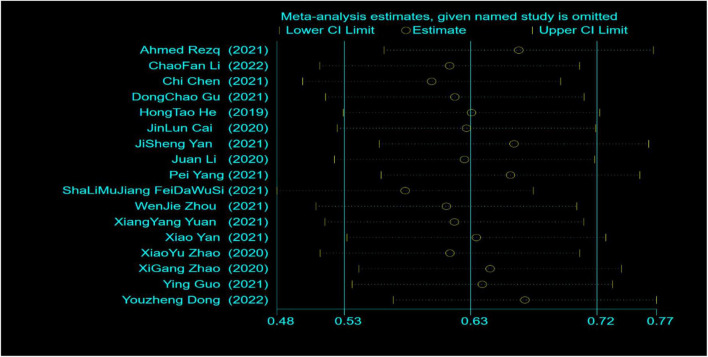
Influence analysis.

**FIGURE 5:**
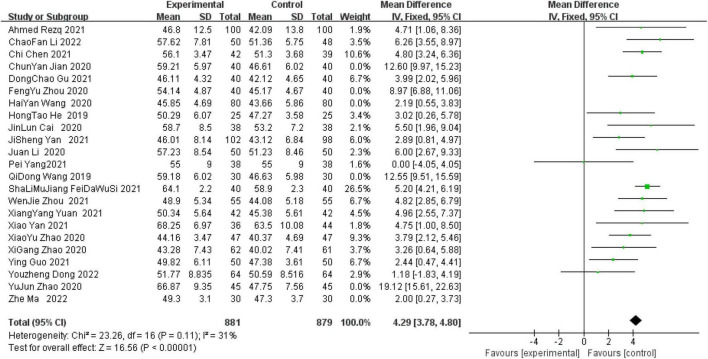
Left ventricular ejection fraction (LVEF) within 6 months after surgery.

A total of 23 studies reported LVEF within 6 months after surgery. Through systematic analysis, it was found that there was a high degree of heterogeneity among the studies (*I*^2^ = 88%, *P* < 0.00001). Therefore, through further sensitivity analysis (Figure 4), we found that Zhou (17), Wang (18), Jian (23), Wang (25), Zhao (34), and Ma (35) were the main sources of heterogeneity. The weights of the studies involving greater heterogeneity were zeroed, and the results of further heterogeneity analysis showed that the heterogeneity among the remaining 17 studies was not statistically significant, so a fixed model was used for statistical analysis. Compared with the control group, the combined use of reperfusion measures and sacubitril-valsartan can significantly improve the LVEF value of STEMI patients at 6 months after surgery. (OR = 4.29, 95% CI: 3.78 to 4.80, *P* < 0.00001) (Figure 5).

### Secondary clinical outcomes

**FIGURE 6:**
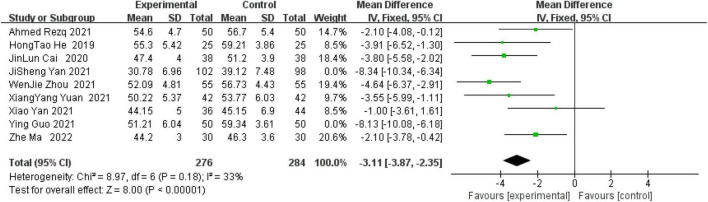
Left ventricular end-diastolic diameter (LVEDD) within 6 months after surgery.

A total of nine studies performed ultrasonographic assessment of LVEDD within 6 months after surgery. A systematic analysis of nine studies found that there was a high degree of heterogeneity among the studies (*I*^2^ = 84%, *P* < 0.00001). Therefore, a sensitivity analysis of the nine studies concluded that JiSheng Yan (29) and Ying Guo (32) were the main sources of heterogeneity. The two studies with large heterogeneity were weighted to zero and the systematic analysis showed that the heterogeneity among the remaining seven studies was not statistically significant, so a fixed model was used for statistical analysis. Sacubitril-valsartan in combination with sacubitril-valsartan significantly improves LVEDD values after myocardial reperfusion compared with controls (OR = −3.11, 95% CI: −3.87 to−2.35, *P* < 0.00001) (Figure 6).

**FIGURE 7:**
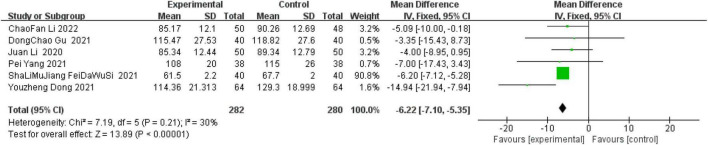
Left ventricular end-diastolic volume (LVEDV) within 6 months after surgery.

A total of six studies performed ultrasonographic assessment of LVEDV within 6 months after surgery. A systematic analysis of nine studies indicated that the heterogeneity among the studies was not statistically significant (*I*^2^ = 30%, *P* = 0.21), so a fixed model was used for statistical analysis. Combined use of sacubitril-valsartan in STEMI treated with reperfusion measures significantly improved LVEDV values after myocardial reperfusion compared with controls (OR = −6.22, 95% CI: −7.10 to −5.35, *P* < 0.00001) (Figure 7).

### Publication bias

**FIGURE 8:**
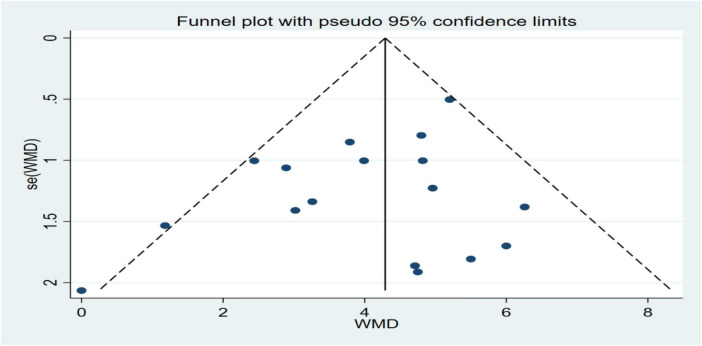
Left ventricular ejection fraction (LVEF) funnel plot at 6 months post-operatively.

**FIGURE 9:**
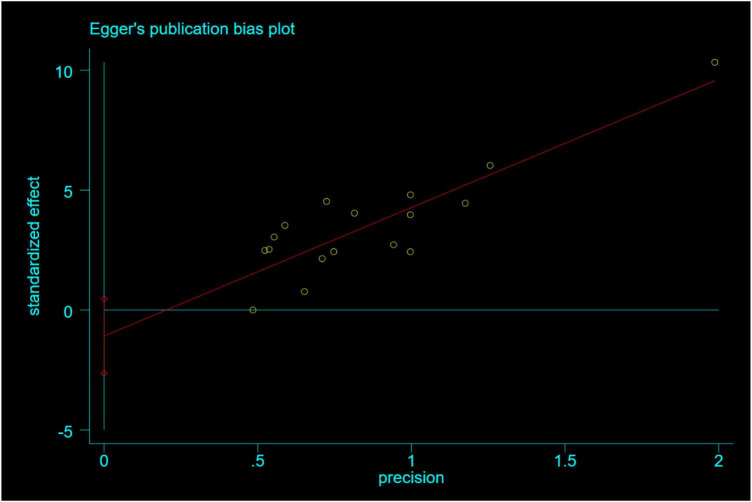
Left ventricular ejection fraction (LVEF) egger’s test within 6 months after surgery.

A total of 23 studies were included, of which 17 reported LVEF values within 6 months after surgery. The funnel plot was used to qualitatively assess no publication bias (Figure 8), and Egger’s linear regression was used to obtain *P* = 0.16. The quantitative assessment of Egger’s linear regression showed no significant publication bias (Figure 9). Because the number of studies involved in both secondary outcomes was less than 10, no publication bias test was performed.

## Discussion

ST-segment elevation myocardial infarction is a moderate, acute and serious disease of cardiovascular disease (36). In the face of STEMI patients, the most effective treatment measure is reperfusion treatment (37). Timely and effective reperfusion measures can effectively reduce the death of myocardial cells, reduce the area of myocardial infarction, especially curb cardiac failure, regulate cardiac function, restore cardiac function, improve the prognosis of patients, and achieve the goal of reducing the mortality and disability rate (38). These reperfusion measures include coronary stent implantation, coronary artery bypass grafting and fibrinolysis (39). The studies involved in our manuscript are all early PCI. Acute ischemia, hypoxia and MIRI of cardiomyocytes will promote the compensatory hypertrophy of surviving cardiomyocytes and form myocardial scars, leading to ventricular remodeling and seriously affecting cardiac function (40). Thanks to the popularity of chest pain knowledge in the world, the rate of culprit vessel opening in STEMI patients is increasing year by year, and the mortality rate of patients in hospital is significantly decreased, however, effective measures to reduce Miri treatment still need to be developed and studied. There has not been a systematic review of sacubitril-valsartan on MIRI to whom it is a novel agent, which is needed for this meta-analysis.

Sacubitril-valsartan has been regarded as one of the most important advances in the field of Cardiology in the past 10 years, and is a new tool of diagnosis and treatment for heart disease (41). As early as 2014 McMurray (42) showed that its more traditional medicine could significantly improve the prognosis of cardiac function in patients, which is supported by its unique dual neuroendocrine regulatory mechanism. In addition, multiple other studies have also shown that sacubitril-valsartan has great promise in the treatment of cardiovascular diseases such as coronary atherosclerotic heart disease, hypertension, and arrhythmias (7, 43, 44). The above research synthesis indicated that sacubitril valsartan improved cardiac function mainly through the type I receptor of angiotensin II with RAAS. Heart failure is one of the most serious complications of STEMI, and the adoption of effective measures to improve cardiac function after reperfusion for STEMI can greatly improve patient outcomes.

The most immediate post-operative MIRI outcome was the LVEF. In Salim’s (45) review was mentioned that reduced LVEF is associated with increased mortality with AMI. Meanwhile, a multicenter study involving 4,495 patients with AMI, in whom LVEF was measured, found that AMI was accompanied by reduced LVEF values and a corresponding increase in mortality (46). There were 794 patients with heart failure enrolled in the study, treated with sacubitril-valsartan during the disease course, and the mean LVEF value increased from 28.2% at the time of the most admission to 37.8% after 12 months of follow-up in all patients (47). Furthermore, in a randomized experiment, sacubitril-valsartan group significantly improved the LVEF value of heart failure patients compared with the control group (LVEF 12.8 ± 12.9 vs. 9.3 ± 12.6%) (48). In addition to that, a retrospective study of 501 patients with heart failure reported a significant increase in cardiac LVEF values from the initial 29.7 ± 4.4 to 40.8 ± 10.4% after sacubitril valsartan administration (49). Simultaneously, Raccah et al. (50) analyzed the clinical effect of patients with Duchenne muscular dystrophy after anti remodeling heart treatment, and found that the total mortality rate of these patients after treatment had a downward trend (OR = 0.36, CI: 0.1 to 1.25, *p* = 0.107). LVEF in the treatment group was significantly improved, with an average difference of 3.77%. In our study, it was found that LVEF was significantly improved in the sacubitril group compared with the non-sacubitril group in patients with STEMI who received reperfusion therapy (OR = 4.29, 95% CL: 3.78 to 4.80, *P* < 0.0001). This indicates that the cardioprotective effect of the combination of sacubitril-valsartan in STEMI patients undergoing reperfusion therapy is obvious.

Studies have shown that patients with heart failure will have significantly elevated LVEDD (51). Study shows outcomes of ventricular remodeling, increased LVEDV in STEMI patients (52). Gu et al. (53) recruited 336 patients with heart failure who were treated with sacubitril-valsartan and showed significantly lower LVEDD after 6 months of sacubitril/valsartan compared to admission levels (59.97 vs. 54.70 mm). In addition, Yin et al. (54) conducted a meta-analysis on the efficacy and safety of pro-urokinase injection during PCI in patients with ST segment elevation myocardial infarction. It was found that compared with the control group, the incidence of MACE and the area of myocardial infarction in the treatment group were reduced, including LVEDD (OR = −0.13, CI: −0.17 to −0.09, *p* < 0.00001). In another prospective study, including 125 patients with heart failure, after the use of sacubitril-valsartan in the treatment phase, there was a significant improvement in LVEDV compared with the initial (206 ± 71 vs. 197 ± 72 mL) (55). Kawarada et al. (56) studied the cardiac function of patients with atherosclerotic renal artery disease with or without heart failure after stent implantation. They found that the mortality rate decreased 6 months after stent implantation, LVEF, left atrial volume index, left ventricular end systolic volume and left ventricular mass index improved significantly. LVEDV was 89.3 ml ± 41 at enrollment, and 86.2 ± 31.2 ml at 6 months after stent implantation. All the above studies support the meta-analysis results of these two indicators. Heart failure is the main outcome of MIRI, so this positive result is clinically important.

Based on the results of this meta-analysis, we have reason to speculate that the combination of sacubitril-valsartan and reperfusion therapy can effectively improve the outcome of STEMI patients. sacubitril-valsartan can exert its protective effect on the heart through a variety of mechanisms. We speculate that because sacubitril-valsartan is a dual inhibitory drug, it can’t only regulate the type I receptor of angiotensin II by targeting, but also regulate RAAS (57). It is well known that the natriuretic peptide family is an important neuroendocrine hormone that regulates water balance and electrolyte balance in the body, and it mainly includes BNP, C-type natriuretic peptide (CNP) and atrial natriuretic peptide (ANP) (58). It exerts cardiovascular protection through mechanisms of kidney (natriuretic, diuretic), blood vessel (vasodilator) and hormone (cortisol, antidiuretic hormone), thus regulating salt and fluid balance, natriuretic peptide also has good metabolic effects, increases lipolysis and insulin sensitivity, and has anti-inflammatory properties (59). In the blood examination of patients with AMI, the expression level of NEPI is significantly increased (60). NEPI is a bioactive protease that can degrade many endogenous vasoactive peptides including natriuretic peptide, adrenomedullin bradykinin, substance P, calcitonin gene-related peptide and so on (2). STEMI patients will show cardiac insufficiency due to the increase of NEPI expression level (61). Interestingly, sacubitril is transformed into the NEPI inhibitor LBQ657 through body metabolism, thus significantly inhibiting the decomposition of natriuretic peptide and alleviating myocardial edema, inflammation and heart remodeling in MIRI (5). In addition, the main role of RAAS is also involved in regulating the water electrolyte balance and maintaining the relative stability of the human body environment (62). Renin and aldosterone are important components of RAAS. Renin is an acidic protease, and its channel of entering the blood is mainly through the renal vein. After entering the blood, renin catalyzes angiotensin I in the blood and converts angiotensin I into angiotensin II under the action of angiotensin converting enzyme (63). The main function of angiotensin II is to increase blood pressure by tightening blood vessels and stimulating the adrenal gland to secrete aldosterone, thereby further increasing the reabsorption of sodium ions and the secretion of potassium ions in human blood (64). If the secretion of angiotensin II is abnormal, it will cause oxidative stress and inflammatory reaction, which has important significance in the progression of atherosclerosis and cardiovascular disease (65). After receiving reperfusion treatment, the myocardial blood supply of STEMI patients is effectively restored, and the occurrence of Miri is also induced. In Miri, the expression level of serum renin will be up-regulated, and the abnormal secretion of renin will lead to the secretion disorder of angiotensin II (66). Valsartan can block the binding of angiotensin II and its receptor, inhibit the action of angiotensin II, expand blood vessels, excrete sodium and diuresis, inhibit sympathetic nerve excitation, and improve hemodynamics (67). Based on the above research results, we can find that salkubatrovalsartan has a significant positive effect in reducing MIRI.

## Conclusion

According to the results of this meta-analysis, the LVEF, LVEDD, and LVEDV of the heart of patients with STEMI were evaluated within 6 months after receiving treatment, which all suggested that salkubatrovalsartan could improve the cardiac function of patients with STEMI. These results show that the combination of sacubitril-valsartan and reperfusion therapy can effectively improve the outcomes of STEMI patients. This treatment is a promising, low-cost, good effect and convenient measure to intervene MIRI for STEMI patients. Although there may be some publication bias, the significant improvement of these indicators is convincing. However, the mechanism of cardioprotective effect of sacubitril-valsartan and whether it can be used as a supplement to the standard reperfusion treatment regimen need further study.

## Limitations

Although the results of egger’s test and Begg test in this meta-analysis show no statistical significance, the possibility of publication bias still exists. In addition, the number of literatures included in this paper is limited, which is mainly because the treatment of MIRI with sacubitril-valsartan is still in a relatively novel stage. Therefore, more high-quality randomized controlled trials need to be carried out to further evaluate the efficacy and safety of sacubitril-valsartan when patients with acute STEMI receive reperfusion. In addition, the small sample size of multiple studies may be the main reason for the heterogeneity between the negative results of some studies and the literature included in the study. Therefore, higher quality and larger sample size RCT results are called for.

## Data availability statement

The original contributions presented in this study are included in the article/supplementary material, further inquiries can be directed to the corresponding author.

## Author contributions

DZ, HW, DL, YuL, GZ, QY, and YaL had access to the data and participated in writing this manuscript. All authors contributed to the article and approved the submitted version.
